# QSRA – a quality-value guided *de novo *short read assembler

**DOI:** 10.1186/1471-2105-10-69

**Published:** 2009-02-24

**Authors:** Douglas W Bryant, Weng-Keen Wong, Todd C Mockler

**Affiliations:** 1 Department of Electrical Engineering and Computer Science, Oregon State University, Corvallis, Oregon 97331, USA; 2 Department of Botany and Plant Pathology and Center for Genome Research and Biocomputing, Oregon State University, Corvallis, Oregon 97331, USA

## Abstract

**Background:**

New rapid high-throughput sequencing technologies have sparked the creation of a new class of assembler. Since all high-throughput sequencing platforms incorporate errors in their output, short-read assemblers must be designed to account for this error while utilizing all available data.

**Results:**

We have designed and implemented an assembler, Quality-value guided Short Read Assembler, created to take advantage of quality-value scores as a further method of dealing with error. Compared to previous published algorithms, our assembler shows significant improvements not only in speed but also in output quality.

**Conclusion:**

QSRA generally produced the highest genomic coverage, while being faster than VCAKE. QSRA is extremely competitive in its longest contig and N50/N80 contig lengths, producing results of similar quality to those of EDENA and VELVET. QSRA provides a step closer to the goal of de novo assembly of complex genomes, improving upon the original VCAKE algorithm by not only drastically reducing runtimes but also increasing the viability of the assembly algorithm through further error handling capabilities.

## Background

Recently the prevalence of high-throughput sequencing technologies has prompted the emergence of a new class of *de novo *sequence assembler. This new class of assembler is specialized to deal with the short, non-perfect reads produced by modern high-throughput sequencers such as those produced by the Illumina and Applied Biosystems SOLiD platforms and includes applications such as SSAKE [1], Velvet [2], EDENA [3], SHARCGS [4], VCAKE [5], ALLPATHS [6], and Euler-SR [7].

Current short-read assemblers all provide the means of stitching together small fragments of DNA sequences to form longer contigs. Common to all short-read assemblers is the concept of bridging overlapping DNA fragments, best expressed by De Bruijn graphs [8]. While all short-read assemblers share this foundation, algorithms differ from assembler to assembler, resulting in significant runtime and output differences.

High-throughput sequencing technologies produce non-negligible error rates in their sequenced output. As such, *de novo *assemblers should be designed to minimize the incorporation of errors during contig extension to ensure that their output is of the highest possible quality. While assemblers unable to account for error may be able to assemble error-free reads, such assemblers fail when working with real-world, non-perfect data.

Our algorithm, QSRA (Quality-value-guided Short Read Assembler), builds directly upon the VCAKE algorithm. Implemented in C++, we show that QSRA not only provides for much faster assembly than VCAKE, but through the use of quality-values QSRA can continue contig extension where VCAKE cannot, resulting in longer average contig lengths. We have also implemented the option to output suspected repeated regions to a separate file, aiding in repeat-related analysis.

## Implementation

### Material

Illumina data generated by shotgun sequencing of the *Pinus pinaster *(pina) and *Pinus gerardiana *(gera03) chloroplast genomes were used for test assemblies [9]. Pina data consisted of 57,586,419 bases with a reference genome of 120,229 bases, resulting in an average coverage depth of 479×. Gera03 data consisted of 44,111,925 bases with a reference genome of 117,306 bases, resulting in an average coverage depth of 376×, with both data sets having an input read length of 33 bp.

One lane of sequence data from the Illumina 1 G Genome Analyzer, generated by shotgun sequencing of the *Streptococcus suis *(suisp) bacterial genome, was also used for test assembly. Produced by the *S. suis *Sequencing Group at the Sanger Institute, this Illumina data [10] consisted of 98,149,464 bases with the reference genome [11] containing 2,007,491 bases, resulting in an average coverage depth of 49×. Read length in this case was 36 bp.

Genome coverage estimates were obtained by alignment of the contigs output by the assemblers against the respective reference genome [9,11] using BLAT [12]. BLAT output was analyzed by comparing the total number of bases in the reference genome with the number of genomic bases uniquely "hit" by assembled contigs. Thus, any contig which BLAT, using the default value of 90% identity, could not match to the appropriate reference genome did not contribute to coverage calculations.

### Algorithm

**Figure 1:**
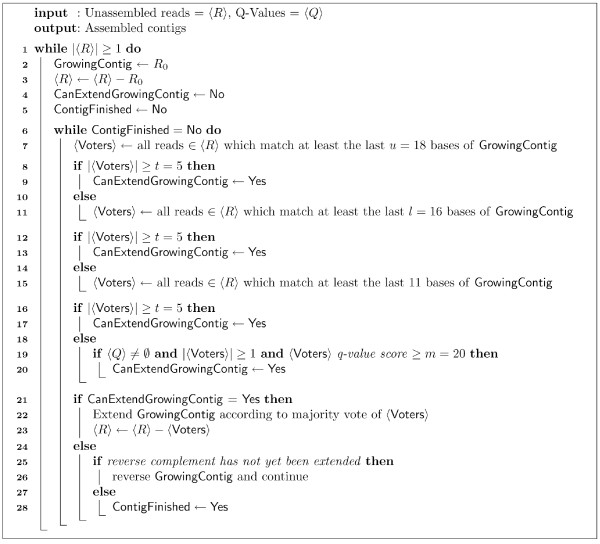
**QSRA Algorithm**. The basic QSRA algorithm, using default values for user-defined parameters. As illustrated, each iteration of contig extension (line 22) is contingent on there existing sufficiently many matching reads, <**Voters**>, from the input set, <*R>*. Population of <**Voters**> is accomplished through a series of weakening conditions. Further algorithmic details are provided in the text.

Figure 1 outlines the basic QSRA algorithm, with details described here. QSRA begins its assembly process in a manner identical to SSAKE by creating a hash table and prefix tree. The hash table stores key-value pairs where the keys are formed by the actual DNA sequence and the values are the number of occurrences of that sequence. The prefix tree contains the unassembled reads as well as their reverse complements, all indexed by their first 11 bases. These data structures hold the unassembled input reads, provided by the user, referred to as <*R*> and <*Q*> in Figure 1. Then, like VCAKE, QSRA finds all *n *of the *k*-mer reads which exactly match the 3' end of the seed (now the growing contig) down to a minimum number of matching bases, *u*, which is a user-defined parameter. QSRA finds these matching reads by searching the prefix-tree data structure. Each matching read found is stored in a linked list along with the number of times each it and its reverse complement occurred in the set of input reads, as well as the number of bases in the read which match to the growing contig. This last measure indicates the position at which bases cease to overlap the growing contig in each matching read and is updated each time the growing contig is extended.

After all exact-matching reads, according to the above criteria, have been added to the linked list, QSRA computes the total number of matches, accounting for duplicated reads such that each copy of a read counts as matching. If this number is less than a user-defined value *t*, QSRA proceeds to find all matching reads down to a lower user-defined limit *l*. If, after this step, *t *matches are still not found, QSRA finds all matching reads down to an overlap of only 11 bases, lines 8–15 in Figure 1.

At this point our algorithm makes its first major departure from VCAKE with one additional and important extension condition. If *t *matching sequences are still not found, and the quality-values (q-values) option is not being used in the current assembly, then extension on that side of the growing contig is halted. However, if q-values are being used in the current assembly, QSRA will extend the growing contig even if *t *matches are still not found, as long as a minimum user-defined q-value score, *m *is met or exceeded at the current over-hanging base position, lines 19–20 in Figure 1. This allows for extension of contigs in some cases far beyond the VCAKE algorithm.

Next the sequences populating the linked list are considered. Each read represented in the linked list casts a number of votes equal to its multiplicity for which base should be added to the end of the growing contig. The base with the highest number of votes is then added to the 3' end of the growing contig, as long as the base garners a user-defined minimum percentage, *c*, of the vote. If the winning base does not hold *c *percentage of the vote, or if the number of votes exceeds a user-defined maximum, *x*, extension is terminated for that side of the growing contig due to assumed sequence repeats. With QSRA sequence repeats can be further analyzed if desired. When a contig's extension is aborted due to a sequence repeat condition and the user has enabled the printing of repeated regions, *r*, the suspected repeated region is output to a separate multi-FASTA format file. All reads that cast a vote at least once are removed from the hash-table and prefix-tree data structures.

The growing contig is extended in this way one base at a time until some condition causes the extension to halt. Extension is then performed on the reverse complemented growing contig in the same manner. When extension is halted on the reverse complemented growing strand, the resulting contig is output to a file in multi-FASTA format and a new seed is selected. This process continues until there are no more reads in the hash-table and prefix-tree.

## Results

All tests were conducted on a 3.0 GHz Xeon Linux machine with 32 GB of RAM. We ran five tests for each of our three datasets, including SSAKE, VCAKE, VELVET, EDENA, QSRA without q-values, and QSRA with q-values, with results shown in Table 1. In all cases the minimum contig length considered in analysis was 1 + read length, with each application set to output contigs of at least this length. In this way singletons were not included in analysis, and all applications were tested on the same basis.

**Table 1:** Results of assemblies of actual Illumina sequencing data on 3.0 GHz Xeon processor with 32 GB memory.

**Organism**	**Error rate (%)**	**Depth**	**Program**	**Program version**	**Program release date**	**Maximum RAM used (GB)**	**Run time (s)**	**Genome covered (%)**	**Largest contig (bp)**	**N50 (bp)**	**N80 (bp)**	**Number of Contigs**	**Runtime options**
pina	5.33	479	SSAKE	3.2	2008	0.91	3463	79.8	3051	241	N/A	24686	-m 16
pina	5.33	479	VCAKE	1.0	05/2007	0.74	8400	68.1	1721	101	N/A	188778	-k 33 -o 34
pina	5.33	479	VELVET	0.6.04	03/2008	0.36	74	58.5	3076	285	N/A	464	-min_contig_lgth 34
pina	5.33	479	EDENA	2.1.1	2008	0.24	210	77.8	3329	400	N/A	3377	-c 34
pina	5.33	479	QSRA	06032008	06/2008	0.84	1553	93.1	1046	94	86	32473	-k 33 -o 34
pina	5.33	479	QSRA*	06032008	06/2008	0.91	1301	99.3	1771	85	85	83004	-k 33 -o 34

gera03	5.18	376	SSAKE	3.2	2008	0.78	1936	85.1	3613	347	42	18093	-m 16
gera03	5.18	376	VCAKE	1.0	05/2007	0.64	3114	82.6	1964	157	96	175451	-k 33 -o 34
gera03	5.18	376	VELVET	0.6.04	03/2008	0.32	55	60.2	4296	386	N/A	311	-min_contig_lgth 34
gera03	5.18	376	EDENA	2.1.1	2008	0.16	98	88.9	3285	535	41	1977	-c 34
gera03	5.18	376	QSRA	06032008	06/2008	0.69	733	99.1	3012	71	71	21132	-k 33 -o 34
gera03	5.18	376	QSRA*	06032008	06/2008	0.75	641	99.0	3012	569	167	60584	-k 33 -o 34

suisp	2.26	49	SSAKE	3.2	2008	2.21	5941	95.8	6475	1036	355	15632	-m 16
suisp	2.26	49	VCAKE	1.0	05/2007	1.66	7202	99.0	11894	1577	718	487006	-k 36, -o 37
suisp	2.26	49	VELVET	0.6.04	03/2008	0.74	144	96.4	18690	4401	1992	1185	-min_contig_lgth 37
suisp	2.26	49	EDENA	2.1.1	2008	0.48	357	97.3	8829	1836	759	3254	-c 37
suisp	2.26	49	QSRA	06032008	06/2008	1.89	3329	96.9	11934	2432	259	18834	-k 36 -o 37
suisp	2.26	49	QSRA*	06032008	06/2008	2.18	3628	98.5	11934	2370	259	168464	-k 36 -o 37

Five tests were run for each of three data sets, including SSAKE, VCAKE, VELVET, EDENA, QSRA without q-values, specified simply as QSRA in Table 1, and QSRA with q-values, specified as QSRA* in Table 1. In addition to the runtime options listed for VELVET, each VELVET run used a tile size of 19 and a coverage cutoff of 5. Only contigs were used, discarding unextended singletons, in the calculation of coverage, N50, and N80 values. Coverage values were determined though analysis of BLAT output by comparing the total number of bases in the reference genome with the number of bases uniquely "hit" by the BLAT alignments with assembled contigs. Thus, any contig which BLAT, using the default value of 90% identity, could not match to its reference genome did not contribute to coverage calculations. N50 and N80 values are equal to the largest contig in the output such that it and all contigs of greater length accounted for 50%/80% of total genome coverage. For *S. suis*, 43.8% of the 36 mer Illumina reads in the data set matched perfectly to the reference genome, which corresponds to an estimated average error rate per sequence base of 2.26%.

For assembly of the pina Illumina reads we see that assembly with QSRA, both with and without q-values, resulted in a drastically shorter running times than VCAKE, at 1,301s and 1,553s respectively, compared to VCAKE's 8,400s. Assembly with QSRA yielded the highest genomic coverage as well, at 99.3% and 93.1% with and without q-values respectively. While EDENA and VELVET yielded the longest individual contigs at 3,329 bp and 3,076 bp, QSRA yielded the next longest contigs at 1,771 bp with q-values and 1,046 bp without q-values.

On the gera03 Illumina reads, QSRA again finished far more quickly than VCAKE at 641s and 733s as compared to VCAKE's 3,114s. Again assembly with QSRA maintains the highest genomic coverage at 99.0% and 99.1%, although in this case VELVET yielded the longest single contig at 3,706 bp, compared to QSRA with its longest contig at 3012 bp.

For the *S. suisp *data set all applications tested performed very well in overall coverage. VELVET yielded the longest single contig at 18,690 bp followed by QSRA's 11,934 bp. QSRA finished in roughly half the time as VCAKE, yielding N50 lengths second only to VELVET.

## Conclusion

For our tests, EDENA and VELVET generally yielded the longest contigs while QSRA generally produced the highest genomic coverage. QSRA was much faster than VCAKE while implementing the use of q-values, while providing the option to output suspected repeated regions. QSRA is extremely competitive in its longest contig and N50/N80 contig lengths output, producing results of similar quality to those of EDENA and VELVET, while finishing in far less time than VCAKE.

Assemblers such as EDENA and VELVET summarize their input reads by constructing a graph which is then processed and traversed in a second step to generate contigs. This processing results in some information loss as input reads are not considered individually as they are in assemblers such as SSAKE, VCAKE, and QSRA. This algorithmic difference is responsible for the trade-off observed here whereby EDENA and VELVET output generally longer but fewer contigs and have lower overall genomic coverage, while SSAKE, VCAKE, and QSRA output generally a higher number of shorter contigs that, in the case of QSRA, result in a higher overall genomic overage.

QSRA provides a step closer to the goal of *de novo *assembly of complex genomes, improving upon the original VCAKE algorithm by not only drastically reducing runtimes but also by increasing the viability of the assembly algorithm by addition of further error handling capabilities.

## Availability and requirements

• Project name: QSRA – Quality-value guided short read assembler

• Project home page: 

• Operating system: UNIX

• Programming language: C++

• Other requirements: None

• License: Free for educational and public use

• Any restrictions to use by non-academics: Must obtain permission from author for non-academic/non-public use

## Abbreviations

The following abbreviations have been used: RAM: Random Access Memory; GB: Gigabyte; GHz: Gigahertz.

## Authors' contributions

DWB coded the QSRA algorithm, conducted the analyses, analyzed the data, and drafted the manuscript. WKW and TCM participated in the design of the study and helped to draft the manuscript. All authors read and approved the final manuscript.
